# Supplementary material for: Identification and functional analysis of non-coding regulatory small RNA FenSr3 in Bacillus amyloliquefaciens LPB-18
Source: PeerJ. 2023 May 15;11:e15236. doi: 10.7717/peerj.15236 (PMC10194069; doi:10.7717/peerj.15236)
Supplement: Supplemental Information 4 [file peerj-11-15236-s004.zip › KO/CK-vs-T1_map/map04066.html]

KEGG PATHWAY: HIF-1 signaling pathway - Reference pathway


|  |  |
| --- | --- |
| **HIF-1 signaling pathway - Reference pathway** |  |

[
Pathway menu
| Organism menu
| Pathway entry
| Show description
| User data mapping
]

Hypoxia-inducible factor 1 (HIF-1) is a transcription factor that functions as a master regulator of oxygen homeostasis. It consists of two subunits: an inducibly-expressed HIF-1alpha subunit and a constitutively-expressed HIF-1beta subunit. Under normoxia, HIF-1 alpha undergoes hydroxylation at specific prolyl residues which leads to an immediate ubiquitination and subsequent proteasomal degradation of the subunit. In contrast, under hypoxia, HIF-1 alpha subunit becomes stable and interacts with coactivators such as p300/CBP to modulate its transcriptional activity. Eventually, HIF-1 acts as a master regulator of numerous hypoxia-inducible genes under hypoxic conditions. The target genes of HIF-1 encode proteins that increase O2 delivery and mediate adaptive responses to O2 deprivation. Despite its name, HIF-1 is induced not only in response to reduced oxygen availability but also by other stimulants, such as nitric oxide, or various growth factors.

|  |  |  |
| --- | --- | --- |
| Reference pathway | 184% 150% 122% 100% 82% 67% 55% | 图片下载 |
